# Buffalo Academy of Medicine1Read and adopted at the regular meeting January 9, 1894.

**Published:** 1894-05

**Authors:** 


### Society Proceedings.

### BUFFALO ACADEMY OF MEDICINE.[1]

### Section on General Medicine.

#### REPORT OF THE COMMITTEE ON TUBERCULOSIS.

*To the Medical Section of the Buffalo Academy of Medicine :*

Your committee, appointed to consider tuberculosis as an infectious disease and the best methods which may be adopted to prevent its dissemination, respectfully submit the following report :

The question of the communicability having been settled by the work of Koch and his followers, and the active causative agent having been proved to be the bacillus tuberculosis, it remains, first, for us to consider the statistics of the mortality of this disease as compared with the mortality of other diseases, and what bearing these statistics may have upon the communicability of the disease and the prevention of its dissemination ; second, to consider the mode or modes of infection, that is, the material in which the bacillus exists inside and outside of the diseased individual, and the avenues by which it may gain entrance to the economy of the previously healthy individual, and whether there are predisposing causes which render one individual more susceptible to the disease than another ; and third, what means may be adopted to prevent the introduction of the germ into a healthy individual and so prevent the spread of the disease.

As to the statistics of the disease, the excellent presentation of the subject by Dr. Gram, as regards the local death-rate, shows that tuberculosis kills 3,375 out of a total number of deaths from all causes of 34,629.

His figures do not differ materially from those of other observers. Osler says : "The death-rate from phthisis is estimated at 15 per cent. of the total mortality." Flick has studied the distribution of the deaths from tuberculosis in a single city ward in Philadelphia for twenty-five years. His researches go far to show how the infectious agent persists in certain houses : "About 33 per cent. of infected houses have had more than one case. Less than one-third of the houses of the ward became infected with tuberculosis during the twenty-five years previous to

1. Read and adopted at the regular meeting January 9, 1894.

1888. Yet more than half the deaths from this disease in 1888 occurred in those infected houses." "The investigations of Cornet upon the death-rate from consumption among certain religious orders devoted to nursing, give some striking facts in illustration of this. In a review of thirty-eight cloisters, embracing the average number of 4,028 residents, among 2,000 deaths in the course of twenty-five years, 1,320 (62.88 per cent.) were from tuberculosis. In some of the cloisters more than three-fourths of the deaths are from this disease."

A most excellent illustration of the wide distribution of the infective agent is given in the report by H. P. Loomis, that of thirty cases presenting no microscopical evidence of old or recent tubercular disease, the bronchial glands of eight were infective to rabbits.

The statistics thus presented show that tuberculosis is (with the possible exception of syphilis) the most widespread of diseases and is the most fatal. The reason for the widespread existence of the disease is that until 1881 its infectious nature had not been proved, although frequently suspected, and that ever since that time no active measures have been adopted by the profession at large to inform the laity of the infectious nature of the disease, or to prevent its spread in the community, although a few individual physicians have now and then, here and there given such information each to his own clientèle.

The consideration of the subject from this point of view brings us to the second division of our subject—namely, the mode, or modes, of infection. This is brought about only by direct contact of the material containing the bacillus with either an open wound or scratch, or an absorbing mucous surface.

How is this contact brought about ? Inoculation through an abrasion of the skin, or other open wound, is rare among human beings, but may occur in those "whose occupation brings them in contact with dead bodies, or animal products. Demonstrators of morbid anatomy, butchers and handlers of hides are subject to a local tubercle of the skin," from which source, occasionally, general infection takes place. The performance of the rite of circumcision by a tubercular operator has occasionally been a source of infection through a wound. "Other means of inoculation have been described, as the wearing of earrings, wearing the clothes of tuberculous patients, the bite of a tuberculous subject, or inoculation from a cut by a broken spit-glass of a consumptive, and

Czerny has reported two cases of infection by transplantation of skin."

As regards infection through eating meat, Osler says: "The meat of tuberculous animals is not necessarily infective. The results of experiments with the flesh of cows are not in accord. This mode of infection, probably, plays a minor rôle in the etiology of human tuberculosis, as usually the flesh is thoroughly cooked before eating. The possibility, however, must be borne in mind, and it would certainly be safer in the interests of a community to confiscate the carcasses of all tuberculous animals."

As regards infection by milk, the same author says: "The milk of an animal suffering from tuberculosis may contain the virus, and is capable of communicating the disease." "It was formerly thought that the cow must present tuberculous disease of the udder, but Ernst has shown that the bacilli may be present, and the milk be infective in a large proportion of cases in which there is no tuberculous mammitis." "This author states the interesting fact that an owner of a herd, known to be tuberculous, withdrew the milk from market and used it, without boiling, to fatten his pigs, which, almost without exception, became tuberculous, so that the whole stock had to be slaughtered. There is no reason to believe that young children, or even adults, are less susceptible to the virus than the calves or pigs, so that the danger of the disease from this source is real and serious. The great frequency of intestinal and mesenteric tuberculosis in children no doubt finds here its explanation. As noted in Woodhead's analysis of 137 cases of fatal tuberculosis in children, the mesenteric glands were involved in 100."

By far the most common method of infection, however, is through the air by means of the respiratory tract.

"It has been found that the expired breath of tuberculous subjects is not infectious," the sputum, however, contains the virus in enormous amount. If this is kept moist there is no danger of infection from it, unless it is brought into direct contact with an open wound, or mucous surface, as in the act of kissing. If, however, this sputum is allowed to dry, it becomes disseminated through the air as minute particles of dust, and as such may be breathed in by a healthy individual, who thus runs the risk of infection.

All careful investigators agree that this is the most common mode of entrance of the tubercle bacillus into the economy.

Osler says in this connection: "Primary tuberculous lesions are in a majority of all cases connected with the respiratory system. The frequency with which foci are met with in the lungs and in the bronchial glands is extraordinary, and the statistics of the Paris morgue show that a considerable portion of all persons dying by accidents or by suicide, present evidence of the disease in these parts. The post-mortem statistics of hospitals show the same widespread prevalence of infection through the air-passages.

Biggs reports that more than sixty per cent. of his post-mortems showed lesions of *pulmonary* tuberculosis. *In 125 post-mortems* at the Foundling Hospital, New York, *the bronchial glands were tuberculous in every case.*

As regards predisposing causes that may render one individual more susceptible than another to the disease, all that we can say, in the light of our present knowledge of the exciting cause, is that anything that lowers the vitality of the individual renders him more vulnerable to the onslaught of the bacillus, and that of especial importance in this respect is the existence of any habit of life which tends to interfere with proper excretion or proper digestion.

With the indisputable evidence before us that tuberculosis is a communicable disease and that every new case is due to infection from another case existing in a human being, or in one of the lower animals; with the knowledge of the habitat of the agent of infection and of the methods in which, and the various avenues by which, this organism gains entrance to the economy, and with the knowledge of methods by which such entrance may be prevented, it would seem that tuberculosis is a perfectly preventable disease and that we ought to be able to stamp it entirely out of existence.

This brings us to the consideration of the most important of the subdivisions of the question, and the following suggestions are made as presenting a practical method to aid in preventing the spread of tuberculosis.

In the first place, recognizing that tuberculosis may exist in milk and meat, we should insist that there be competent and thorough inspection of dairies and meats, and that all diseased animals should be slaughtered and cremated. For such thorough work it is necessary that the health department should have a thoroughly equipped bacteriological laboratory, and that the city should be willing to pay a sufficiently large salary to insure the employment of a competent bacteriologist.

In the second place, we must recognize the fact that most of the laity do not appreciate that consumption is communicable, and we should lend our energies to the education of the public in this respect.

In the third place, as soon as a diagnosis of tuberculosis has been made in a given case, the attending physician should inform either the patient or some responsible member of the family, of the infectious nature of the disease, and should give explicit directions as to how to prevent its spread.

In the fourth place, physicians should report all cases of tuberculosis to the health department as soon as recognized, just as they do other infectious diseases, and the health department should send, either to the patient or to some responsible member of the family, in the following, or in such form as the health department may deem fit, a printed circular containing the following information :

Tuberculosis, popularly known under the names of consumption, decline, scrofula, marasmus, wasting disease, inanition, lupus, and white swelling, is a contagious disease; that is, every new case is produced by exposure to some other case.

The agent of infection is sometimes found in meat and milk. Therefore, unless there is competent inspection of foods, and the sources are known to be free from disease, milk should be boiled, and meat should be thoroughly cooked before being used as food.

In human beings, the agent of infection exists in the pus (matter) given off in the discharge from tubercular abscess, or from lupus ; in the discharge from the bowels in marasmus or tuberculosis of the bowels; in the urine in tuberculosis of the kidney, bladder or other part of the genito-urinary apparatus ; in whatever may be vomited, but, *most of all*, in the material that is *coughed up and spit out* in consumption of the lungs.

As long as these materials are kept moist there is no danger of infection, unless they are brought into direct contact with the mouth, nose, eyes or other mucous surface, or an open wound or scratch. If they dry on the clothing, on handkerchiefs, in a cup or spittoon, or other vessel, or on the floor, walls or sidewalk, they become disseminated through the air as minute particles of dust. and as such may be breathed in by a healthy person, who thus runs the risk of infection.

Consequently, the underclothing and bedclothing of the consumptive should be changed at least twice a week (more often if the patient sweats much), and those articles of clothing taken off should be boiled for half an hour before they are washed. The movements from the bowels should be passed into vessels containing water, and these vessels, as well as those into which the urine may be passed, should be thoroughly cleaned with boiling water. The sputum should never be expectorated upon the floor, walls or sidewalk, or in stores, street cars or other public place, or into a handkerchief or other cloth upon which it might become dried, but always into a cup or other suitable vessel containing water, which is kept covered when not in actual use. If the patient is walking about, a wide-mouthed bottle, containing some water and securely corked, can be carried in the pocket and used when necessary. The sputum thus collected should be emptied from the vessel three or four times in the twenty-four hours, and immediately burned, boiled or otherwise destroyed. The vessel and its cover or stopper should each time be thoroughly cleansed with boiling water. The table utensils, including the napkin of the patient, should be thoroughly boiled before being otherwise washed. The mouth, nose and throat of the patient should be thoroughly cleansed, at least, twice daily with a cleansing solution made by dissolving one teaspoonful of sodium bicarbonate (saleratus) in one-half pint of warm water.

No musical instrument or other implement which when in use is placed to the lips or in the mouth should be used by any well individual after it has been used by a consumptive, unless it has been thoroughly cleaned and disinfected. The patient should not kiss anyone, nor should anyone kiss the patient on the mouth; and under no circumstances should anyone sleep in the same bed with the patient. The patient's room should be thoroughly ventilated and should never be tightly shut up. It should be as sunny a room as possible. It should be thoroughly disinfected, at least, twice a year during the lifetime of the patient and immediately after his or her death.

Signed,
HENRY R. HOPKINS,
JOHN H. PRYOR,
W. SCOTT RENNER,
ERNEST WENDE,
F. WHITEHILL HINKEL,
DeLANCEY ROCHESTER.

Tuesday, December 12, 1893.

In submitting this report, your committee recommends that a sufficient number of copies be printed, and that a copy be sent to every physician practising in the City of Buffalo, that a copy be sent to the Health Department of the City of Buffalo, to the State Board of Health, to the national health authorities at Washington, to the New York Academy of Medicine and to the medical press of Buffalo, New York, Boston, Philadelphia and Chicago, and to the general press of Buffalo.

